# The Lingering Shadow: Long-Term Effects on COVID-19 Survivors

**DOI:** 10.3126/nje.v15i1.83967

**Published:** 2025-09-02

**Authors:** Brijesh Sathian, Javed Iqbal, Padam Simkhada, Hanadi Al Hamad

**Affiliations:** 1 Geriatrics and long-term care department, Rumailah Hospital, Hamad Medical Corporation, Doha, Qatar; 2 Nursing Department, Hamad Medical Corporation, Doha, Qatar; 3 School of Human and Health Sciences, University of Huddersfield, Huddersfield, United Kingdom

## The Hidden Pandemic After the Pandemic

As the world emerges from the acute phase of the COVID-19 pandemic, a quieter but no less devastating crisis persists: the long-term effects on millions of survivors. What initially presented as a respiratory infection has transformed into a complex health challenge known as “Long COVID” or post-acute sequelae of SARS-CoV-2 (PASC). This condition affects approximately 65 million people worldwide, with symptoms persisting for months or even years beyond the initial infection [1]. Far from being a minor afterthought, Long COVID marks a significant shift in our understanding of viral diseases and their lasting impacts on health, society, and economies. It urgently calls for attention from policymakers, healthcare professionals, and researchers to alleviate the persistent burdens carried by survivors.

## Physical Burden: Multi-Organ Impact of Long COVID

The physical symptoms of Long COVID are wide-ranging and incapacitating, involving almost every organ system. Fatigue and weakness are the most common, impacting up to 28% of survivors one year after infection, often accompanied by shortness of breath in 18% of cases [2]. Respiratory complications, including abnormal chest CT findings and diminished lung function, persist in over 70% of intensive care unit (ICU) survivors, causing chronic breathlessness that hampers everyday activities [3]. Cardiovascular issues are equally concerning, with many patients facing increased risks of heart failure, arrhythmias, stroke, and thrombosis, sometimes resulting in lasting damage to vital organs such as the heart, lungs, and kidneys. Studies reveal that 59% of survivors suffer from single-organ damage, while 27% experience multi-organ involvement even after a year. Metabolic disturbances like new-onset type 2 diabetes further complicate recovery, demonstrating the systemic nature of the virus’s effects [4].

## Neurological and Cognitive Consequences

Neurological and cognitive symptoms are central to Long COVID, often described as “brain fog.” Memory impairment, difficulty concentrating, and cognitive decline affect 19% and 18% of survivors at one year respectively—effects comparable to accelerated aging or mild intoxication [1]. These dysfunctions arise from processes including neuroinflammation, endothelial damage, and persistent viral presence in the brain. Severe cases involve persistent symptoms such as paresthesia, dizziness, and balance disorders, which significantly impair survivors’ ability to work or engage socially. Gastrointestinal problems, including nausea and altered gut microbiota, last at least 14 months in some, linked to dysbiosis and ongoing inflammation. Reproductive health is also affected, with menstrual irregularities among women and erectile dysfunction among men adding to the physical toll [1].

## Psychological and Psychiatric Dimensions

Mental health impacts represent some of the most challenging consequences, intertwining closely with physical symptoms and creating a vicious cycle. Anxiety affects 22% of survivors, depression 23%, and insomnia 12% one-year post-infection, with higher incidences in those hospitalized [2]. ICU survivors face even greater challenges, with anxiety reported by 48%, depression by 23%, and post-traumatic stress disorder (PTSD) by 25%, often triggered by nightmares and isolation during treatment [3]. The mental health impact extends to family members as well, with 66% experiencing anxiety and 55% PTSD, highlighting the ripple effect on households [3]. In parts of Central and Eastern Europe, survivors report significant disruptions to work, family, and social life, aggravated by symptoms such as brain fog and diminished lung capacity [5]. Prevalence data underscores a grim reality: at least 45% of all survivors continue to experience symptoms such as fatigue (25-35%), breathlessness (18-20%), and sleep problems (15-23%), with women and initially severe cases at greater risk [6].

## Societal and Economic Implications

Long COVID poses a substantial societal burden. In the United States alone, between 4.3 and 9.7 million adults suffer from activity-limiting symptoms, disproportionately affecting women and contributing to workforce shortages and healthcare pressures [4]. Globally, its incidence ranges from 10-30% in non-hospitalized cases to 50-70% in hospitalized ones, resulting in significant lost productivity and healthcare costs [1]. Treatment options remain limited; approaches such as pacing for fatigue, beta-blockers for dysautonomia, and antihistamines for inflammation provide symptomatic relief, yet a definitive cure is lacking. Underlying mechanisms, including immune dysregulation, autoimmunity, and microvascular clotting, require focused research, alongside supportive strategies like social support and resilience-building that encourage posttraumatic growth despite adversity [5].

## Conclusion: Confronting the Lingering Shadow

The editorial message is unequivocal: Long COVID cannot be sidelined. Governments must enhance access to multidisciplinary care clinics, fund long-term research, and embed mental health services within recovery programs. Vaccination reduces risk by 10-12%, reinforcing that prevention remains essential [1]. For survivors, recognition is critical acknowledging their struggles and investing in their futures. As research shows, organ damage may be persistent, with long-term effects still emerging. Ignoring this shadow will only deepen the pandemic’s legacy; confronting it directly could reshape resilience in the post-COVID world.
